# Understanding meta-population trends of the Australian fur seal, with insights for adaptive monitoring

**DOI:** 10.1371/journal.pone.0200253

**Published:** 2018-09-05

**Authors:** Rebecca R. McIntosh, Steve P. Kirkman, Sam Thalmann, Duncan R. Sutherland, Anthony Mitchell, John P. Y. Arnould, Marcus Salton, David J. Slip, Peter Dann, Roger Kirkwood

**Affiliations:** 1 Research Department, Phillip Island Nature Parks, Cowes, Victoria, Australia; 2 Department of Environmental Affairs, Oceans and Coasts Research, Victoria and Alfred Waterfront, Cape Town, South Africa; 3 Animal Demography Unit, Department of Biological Sciences, University of Cape Town, Cape Town, South Africa; 4 Department of Primary Industries, Parks, Water and Environment, Hobart, Tasmania, Australia; 5 Department of Environment, Land, Water and Planning, Orbost, Victoria, Australia; 6 School of Biological and Chemical Sciences, Deakin University, Burwood, Victoria, Australia; 7 Department of Biological Sciences, Macquarie University, North Ryde, New South Wales, Australia; 8 Taronga Conservation Society Australia, Mosman, New South Wales, Australia; 9 Wageningen Marine Research, Den Helder, The Netherlands; Sanya Institute of Deep-sea Science and Engineering Chinese Academy of Sciences, CHINA

## Abstract

Effective ecosystem-based management requires estimates of abundance and population trends of species of interest. Trend analyses are often limited due to sparse or short-term abundance estimates for populations that can be logistically difficult to monitor over time. Therefore it is critical to assess regularly the quality of the metrics in long-term monitoring programs. For a monitoring program to provide meaningful data and remain relevant, it needs to incorporate technological improvements and the changing requirements of stakeholders, while maintaining the integrity of the data. In this paper we critically examine the monitoring program for the Australian fur seal (AFS) *Arctocephalus pusillus doriferus* as an example of an ad-hoc monitoring program that was co-ordinated across multiple stakeholders as a range-wide census of live pups in the Austral summers of 2002, 2007 and 2013. This 5-yearly census, combined with historic counts at individual sites, successfully tracked increasing population trends as signs of population recovery up to 2007. The 2013 census identified the first reduction in AFS pup numbers (14,248 live pups, -4.2% change per annum since 2007), however we have limited information to understand this change. We analyse the trends at breeding colonies and perform a power analysis to critically examine the reliability of those trends. We then assess the gaps in the monitoring program and discuss how we may transition this surveillance style program to an adaptive monitoring program than can evolve over time and achieve its goals. The census results are used for ecosystem-based modelling for fisheries management and emergency response planning. The ultimate goal for this program is to obtain the data we need with minimal cost, effort and impact on the fur seals. In conclusion we identify the importance of power analyses for interpreting trends, the value of regularly assessing long-term monitoring programs and proper design so that adaptive monitoring principles can be applied.

## Introduction

In the marine environment, monitoring the abundance and trends of a top predator can provide measures of ecosystem health and management success [1–5]. While population assessments for marine predators are challenging, they are generally easier and more accurate for species that breed on land, such as seabirds and pinnipeds, than for species that are wholly aquatic, such as cetaceans [6–9]. However, challenges remain: the proportion of the population that is ashore can be difficult to determine, breeding colonies can be difficult to access and in some cases, geographically dispersed [10–12]. Trends analyses can be limited by sparse or short-term abundance estimates for populations that can be logistically difficult to monitor regularly over time [13, 14], and few include a power analysis of the trend to understand its reliability [15–17]. An additional consideration is that surveying seabird and pinniped colonies may cause disturbance and potentially reduce breeding success, particularly if a greater frequency of surveys is required to improve the reliability of the trend. Especially when capacity is limited, monitoring may be focused on single sites. The limited spatial coverage of such studies reduces the usefulness of the data for broader applications such as trophic modelling or for informing ecosystem management [1, 18–20]. Ideally, survey design needs to take into account potential variation in population dynamics over space and time (depending on research or monitoring objectives), and ensure compatibility of survey methods over space and time [2, 5, 21].

In the case of fur seals, population estimates are frequently based on the number of live pups that are confined to the breeding colony (hereafter referred to as ‘colony‘) for the first few weeks of life [22]. They are easy to distinguish from older fur seals due to their smaller size, behaviour and dark natal pelage, and the timing of births each year is synchronous and predictable. For example, the median birth date for Australian fur seals (AFS) *Arctocephalus pusillus doriferus* is late November, but colonies may not be accessible because of aggressive breeding bulls until late December or January [23]. Therefore, the year allocated to a breeding season represents the year the season began (e.g. a breeding season that begins in November 2013 and is surveyed in January 2014 is referred to as the 2013 breeding season). Fur seals are important upper trophic level predators that, in Australia, are protected marine species and pose specific management challenges that include fisheries and aquaculture interactions; economic value through tourism; potential impacts on other important marine species such as seabirds; and emergency situations such as oil spills [1, 24–26]. For these reasons, it is important to obtain accurate and relevant abundance information and interpret change in fur seal populations.

Ideally, to interpret population trends and drivers of change, which can be unique to a location or region, the selected index of change (in this case live pups) should be determined annually [2, 27, 28]. Longer intervals between estimates may obscure short-term fluctuations and could delay recognition of changes in the population, as well as factors that influence population change [29]. Performing frequent e.g. annual estimates can be logistically challenging, for reasons such as inconsistent funding or adverse weather and, depending on the technique, can have implications for the welfare of animals. Also, while an index of population abundance is important, it can only detect change and does not allow for an understanding of the causes behind detected change in population. To understand density dependent effects: age-structure, mortality and density are useful parameters; and to understand how the environment may be affecting the population: foraging ecology, animal health and diet can be highly informative [2, 30–33]. In this paper we aim to determine whether the monitoring program for the AFS is achieving its goals: to determine the pup abundance of the AFS and provide trends for the population.

**Fig 1:**
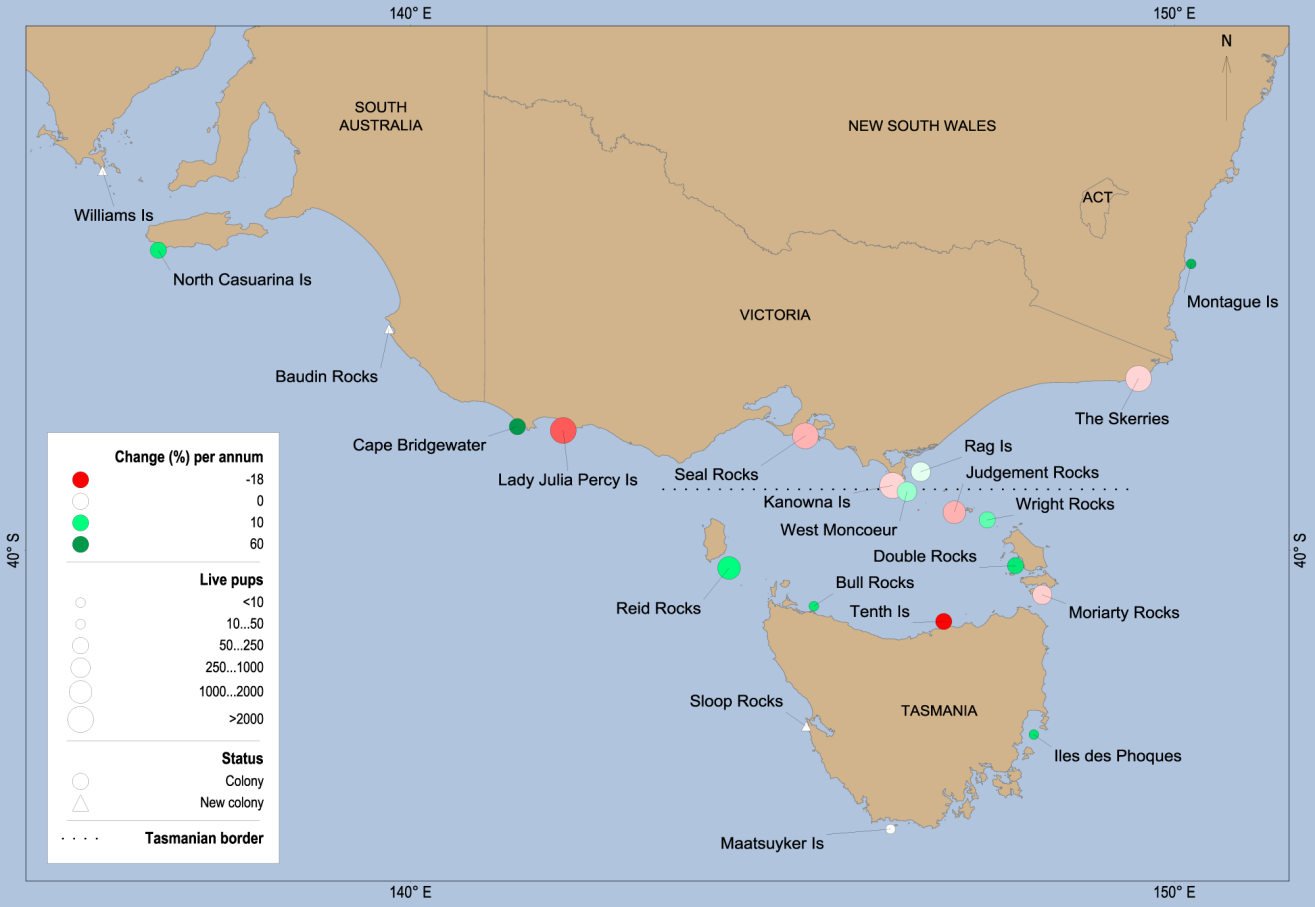
Map showing the range of the Australian fur seal with change (%) per annum between the 2007 census and the 2013 census. Note the pup estimate used for The Skerries and Maatsuyker Island was obtained in the 2014 breeding season and the % change per annum for Iles des Phoques were calculated from the 2002 census because the colony was not visited during the 2007 census. The number of live pups is indicated by the size of the colony shape. “Colonies” represent previously identified locations with pups and “new colonies” are those that were identified during the 2013 census.

In Australia, it is thought that up to 26 AFS colonies existed prior to the onset of commercial harvesting in the early 1800s [34]. The uncertainty regarding the number of colonies is caused by an inability to determine the exact location of all sealing locations and a lack of clarity as to which species was harvested [35]. At the end of commercial harvesting in 1921, fewer than 10 AFS colonies were extant with greatly reduced numbers of fur seals [35]. The number and size of colonies in south-eastern Australia have regenerated subsequently (Fig 1), but with 20 breeding sites identified in 2007 the population is still considered to be in recovery [36].

Monitoring of AFS pup numbers was rare and sporadic up to the late 1960s [37] (Warneke unpub. data) and was then opportunistic and ‘surveillance’ in style until 2002 [34, 38]. At this point, the monitoring program adapted to a coordinated range-wide census performed every five years [39]. The population seemed to double in size between 1986 and 2002, from <10,000 to approximately 22,000 fur seal pups, at a growth rate of 5% per annum [39, 40]. This may have been in response to full legislative protection of the AFS, enacted in 1975. Prior to this, recovery of the population following the end of commercial harvesting in 1921 had been minimal, perhaps to some extent due to on-going lethal interactions with fisheries [40]. There was little overall growth in the population between 2002 and 2007 [36] when the total population was estimated at 120,000 seals, although the breeding range had expanded. It was speculated that the population had approached carrying capacity within the core breeding area of Bass Strait, limited either by breeding areas and/or prey availability, and that this may have contributed to the range expansion [36].

Surveys have used a variety of methods in response to research interest, opportunity and regional or state access to funding [23, 29, 38, 41–45]. The technique employed also varied at colonies over time, but was standardized by site for the censuses performed after 2002. In general, ground counts were used at smaller colonies (<500 pups) or colonies where the terrain was steep and dangerous for handling pups, capture-mark-resight (CMR) at larger colonies (>500 pups), and aerial surveys at colonies that were particularly difficult to access and/or had an open topography [36, 46]. At sites with high pup numbers, or more cryptic and rocky terrain where pups can hide, CMR is preferable because the resulting estimates have higher precision and accuracy than a ground or aerial count [36, 43, 47].

This 5-yearly census program was a great improvement, providing estimates of total abundance at intervals and the detection of positive population trends. However, because annual variability in pup abundance can be large [8] and surveys infrequent, the true rate of increase and the reliability of the trends are unknown. It is generally understood that the ideal long-term monitoring program is adaptive and able to evolve over time [21]. Such a program is resilient and informative with regular review so that it can be modified to maximise success. In this study, we report results of a third range-wide census of pup abundance for the 2013 breeding season, and interpret temporal trends for each breeding colony from the long-term data. Using the updated time series, we estimate the power to detect changes in the trends and critically examine the success of this five-yearly census as a long-term monitoring program. We then provide recommendations to improve our ability to interpret the changes observed in the population and respond adaptively. This paper provides valuable information on how to design monitoring programs for pinnipeds using real data as a case study. We explore changes in the program that will facilitate a transition to an adaptive monitoring program to provide reliable and useful information for managers and stakeholders.

## Materials and methods

In Victoria, the research was performed under animal ethics permit 1.2011 from the Phillip Island Nature Park Animal Ethics Committee and Wildlife Permit 10006785 from the Department of Environment and Primary Industries. In Tasmania, the research was permitted by Department of Primary Industries, Parks, Wildlife and Environment through Standard Operating Procedures for staff.

### Species-wide census of pup production

The AFS has a single annual pupping period in the Austral summer and 90% of pups are born in a 3–4 week period with a peak in early December [23]. Given this breeding synchrony, it is reasonable to assume a closed population at each individual site, with equal likelihood of observing all pups at the time of the surveys since they are of a similar age and at a similar stage of development. This improves the accuracy of the abundance estimate and trend [48, 49].

To continue the five yearly monitoring program for the AFS and with the intention of obtaining temporally and spatially aligned data, a census of live pup numbers was conducted in 2013 across the range of the AFS as described by Kirkwood, Pemberton [36]. Between 2 and 6 replica assessments were performed at each colony. Some sites included sub-locations and mean estimates were calculated per sub-location then summed for a total estimate for the colony and standard errors calculated, repeating methods in Kirkwood, Pemberton [36]. To replicate the 5-year survey interval, we planned the census for the 2012 breeding season but funding constraints meant only Seal Rocks was surveyed (using) CMR in that season. Thus the main census was postponed to 2013. From December 2013 to February 2014, 20 out of 22 recognised colonies (colony descriptions are provided in Table 1). Two colonies, The Skerries and Maatsuyker Island, were not surveyed in 2013 due to a lack of resources but were surveyed one year later, in the 2014 breeding season. Additional surveys were performed at Cape Bridgewater in 2014 and 2015: this site is on mainland Victoria and consists of approximately 100 pups that are estimated via direct count. The simplified logistics of the mainland site at Cape Bridgewater enabled more frequent visits.

**Table 1:** Descriptions of colonies (n = 22) for the Australian fur seal and dates of pup estimates from December 2013 to February 2015.

Colony	Agency	Latitude	Longitude	Area (ha)	Height (m)	Breeding area description	Estimate method	Date of pup estimate
**Victoria**								
Lady Julia Percy Island (LJP)	PINP & DELWP	38°25’S	142°00’E	150	40	Inter-tidal platforms, cobble beaches and caves	CMR	07–10 Jan 2014
Seal Rocks (SR)	PINP	38°30’S	145°10’E	8	10	Cobble beaches and outcrop	CMR	28–30 Dec 2012, 28–30 Dec 2013
Kanowna Island (Kan)	Deakin Uni	39°10’S	146°18’E	130	90	Granite slopes and boulders	CMR	08–09 Jan 2014
The Skerries* (Ske)	PINP & DELWP	37°45’S	149°31’E	8	10	Boulder outcrop, three islets	CMR	19–21 Jan 2015
Rag Island (Rag)	Deakin Uni	38°58’S	146°42’E	3	15	Granite slopes and boulders	Count	20 Jan 2014
Cape Bridgewater (CB)	PINP & DELWP	38°23’S	141°24’E	1	0	Cave and inter-tidal platforms	Count	11 Jan 2014, 15 Jan 2015
**Tasmania**								
Reid Rocks (RR)	DPIPWE	40°14’S	144°09’E	10	8	Series of flat-topped, columnar-dolerite islets	Aerial	19 Jan 2014
West Moncoeur (WM)	DPIPWE	39°14’S	146°30’E	4	30	Steep granite slopes and boulders	Count	19 Jan 2014
Judgment Rocks (JR)	DPIPWE	39°30’S	147°07’E	14	50	Dome shaped, steep, granite, some flat areas	CMR	13–16 Jan 2014
Tenth Island (TI)	DPIPWE	40°57’S	146°59’E	1	8	Single, low basalt islet	CMR	07–08 Jan 2014
Moriarty Rocks (MR)	DPIPWE	40°35’S	148°16’E	4	7	Granite islets (East & West)	Count	20 Jan 2014
Wright Rocks (WR)	DPIPWE	39°36’S	147°33’E	4	30	Dome shaped, steep, granite	Count	17 Jan 2014
Double Rocks (DR)	DPIPWE	40°20’S	147°55’E	1	15	Flat, rectangular, granite	Count	20 Jan 2014
Bull Rock (BR)	DPIPWE	40°44’S	147°17’E	1	5	Columnar jointed basalt	Count	19 Jan 2014
Sloop Rocks (SlR)	DPIPWE	42°18’S	145°10’E	2	15	Granite islets, slopes and boulders	Count	07 Feb 2014
Iles des Phoques (IdP)	DPIPWE	42°25’S	148°09’E	8	7	Granite island	Count	30 Jan 2014
Maatsuyker* (Maat)	DPIPWE	43°38'S	146°17E	186	284	Quartzite	Count	26 Feb 2015
**South Australia**								
Williams Is (WI)	SARDI & SA Museum	35°01’S	135°58’E	141	40	Upper platform of calcarenite laying over on ‘a U-shaped ridge of pink granite	Count	14 Mar 2014
North Casuarina (NC)	SARDI & SA Museum	36°40’S	136°42’E	4	10	Low schist islet, calcarenite cap	CMR	28–29 Jan 2014
Cape Gantheaume	SARDI & SA Museum	35°04’S	136°42’E			Basalt rocky coastline above tidal zone	Incidental obs	Jan 2014
Baudin Rocks (Bau)	SARDI & SA Museum	37°06’S	139°43’E	5	12	Two major islets and at least 17 smaller islets of calcareous sandstone	Count	Mar 2014 (R. Roach, pers. comm.)
**NSW**								
Montague Island (Mon)	Macquarie Uni & Taronga Zoo	36°15’S	150°14’E	81	64	Basalt and granite island with rocky outcrops	CMR	13 Jan 2014

*Censused one year later than other colonies

Acronyms and abbreviations, listed alphabetically: Department of Environment Land Water and Planning (DELWP), Department of Primary Industries, Water and Environment (DPIPWE), Phillip Island Nature Parks (PINP), South Australian Research and Development Institute–Aquatic Sciences (SARDI), South Australian (SA), University (Uni).

### Temporal trends in pup abundance

Live pup numbers at several of the AFS colonies’ were estimated by multiple methods over time (ground count, CMR, or aerial survey). To reduce the variability in the data caused by multiple methods being used at a site across a temporal scale, the predominant method for each site was selected and only data for that method from 1986–2013 were included for each site in the analysis. Data prior to 1986 were unreliable and not included. Eight colonies employed the CMR method, 12 used direct counts and one, aerial survey (Table 2). By only including data of the same method at a site, we reduced the variability caused by different methods.

**Table 2:** The sub-set of live pup data of Australian fur seals per colony and of consistent method, including ground counts (count), aerial surveys (aerial) and capture-mark-resight estimates (CMR). Raw values were cross-checked, some were obtained from individual agencies and published records [29, 36, 38, 39, 42, 44, 45, 52–56]. Year is from the start of that breeding season (Nov-Dec). Full site names and abbreviations are provided below the table. The total number of pups (Total) is provided for the census years in 2002, 2007 and 2013.

Site	LJP	SR	Kan	Ske	Rag	CB	RR	WM	JR	TI	MR	WR	DR	BR	IdP	SlR	Maat	NC	WI	Bau	Mon	
Method	CMR	CMR	CMR	CMR	Count	Count	Aerial	Count	CMR	CMR	Count	Count	Count	Count	Count	Count	Count	CMR	Count	Count	CMR	Total
**1986**							775															
**1987**																						
**1988**																		0				
**1989**							1131	217			234	1					0	0				
**1990**								235			858											
**1991**		2826					885	259			897	1										
**1992**					0			225			665				0			0				
**1993**																					1	
**1994**									1859	354	1035							0				
**1995**						1		373	2365	173	689	3						0				
**1996**							1476		1971	386		3										
**1997**		4794					579	155	2548	277	345	1	0					0				
**1998**							210		2539	364												
**1999**	4867			1867			142	252	2421	287		1		2								
**2000**			1724	2237																		
**2001**														1	1							
**2002**	5899	4882	2301	2486	30	7	259	257	2427		1007	5		7	1						1	17,268
**2003**			2936																			
**2004**			3206																			
**2005**																						
**2006**																					2	
**2007**	5574	5660	2913	2705	277	7	395	204	2387	448	598	130	51	7	0		1	29			2	21,387
**2008**																						
**2009**																						
**2010**																						
**2011**																						
**2012**		3725																74				
**2013**	2659	4092	2429		295	120	1570	256	1710	138	486	187	157	21	10	16		75	2	6	19	14,248
**2014**				2254		95											13					
**2015**						146																

Colony names and abbreviations by state of Australia

**Victoria;** Lady Julia Percy Island (LJP), Seal Rocks (SR), Kanowna Island (Kan), The Skerries (Ske), Rag Island (Rag), Cape Bridgewater (CB).

**Tasmania;** Reid Rocks (RR), West Moncoeur (WM), Judgment Rocks (JR), Tenth Island (TI), Moriarty Rocks (MR), Wright Rocks (WR), Double Rocks (DR), Bull Rock (BR), Sloop Rocks (SlR), Iles des Phoques, (IdP), Maatsuyker (Maat).

**South Australia;** Williams Is (WI), North Casuarina (NC), Cape Gantheaume, Baudin Rocks (Bau).

**New South Wales;** Montague Island (Mon)

Dependable and complete surveys of the total live pup abundance of AFSs were obtained during the three censuses 2002, 2007 and 2013. Three data points over eleven years were not considered a large enough time series for performing trends analysis for the total population combined. Additionally, the reduction in pup numbers during the 2013 census resulted in three highly variable results for the total population that could not be used with confidence. Therefore, the sub-set data with standardized methods for a colony (Table 2) were used to calculate trends separately by colony. This approach enabled the trends to be calculated over a larger temporal scale and the inclusion of data that was obtained outside the three range-wide censuses performed in 2002, 2007 and 2013. It also avoided combining data from colonies that were surveyed by different methods. Using the larger dataset improved the reliability of colony-specific trends. Additionally, trends can be site specific and vary depending on, for example, the maturity of the colony and its density and therefore much insight can be gained by analysing the trends separately [28, 50].

Dead pups were not counted throughout the AFS monitoring program: a major shortfall of the design. Based on mortality rates of AFS pups [45, 51], previous papers reporting census trends have added 15% to the CMR result to estimate total pup production, which is in effect a standardization that does not affect the trend. Correction factors have also been applied for some sites in an attempt to standardise data obtained by different methods and estimate a more accurate total number of live pups [23, 39, 42, 43, 52]. Total population abundance (adults, juveniles and pups) was then calculated by multiplying the total pup production estimate by between 3.5 and 4.5 [1, 10, 36, 39, 45]. Because correction factors and early pup mortality rates can be colony and year specific, total population estimates based on these correction factors are of unknown accuracy. For this paper, we use only the raw data for live pups to perform the analysis.

Generalized Linear Models (GLMs) were applied to the data individually for each colony (live pup estimate ~ year) using the package “MASS” (v7.3–45) [57] in the R statistical environment (v3.1.1, R Core Team, 2013). All GLMs were fitted with a Negative Binomial distribution to correct for over-dispersion (highly inflated *θ)* [58]. The use of a Negative Binomial distribution also avoided the likelihood of standard errors being biased downward, resulting in spuriously large z-values [58]. The Negative Binomial GLM is not suitable for a small sample size, therefore dispersion parameters (*θ)* were provided to assess the confidence in pup abundance trends [58].

### Power analysis of population trend

An *a priori* power analysis using G*Power (version 3.1.9.2, [59, 60]) was used to investigate the ability for surveys conducted at intervals of every three or five years to reliably detect changes in trends over a 30-year duration. This test is typically applied as a survey design tool prior to beginning a monitoring program. The Program G*Power computes the statistical power analyses for z-tests (using the Poisson distribution for count data) and we wanted to detect an effect size of 30% change with 90% confidence (p<0.10) and power of 0.80; this effect size was considered to be realistic and achievable.

To obtain the power of the GLM trends for each individual colony, we performed a *post-hoc* analysis including the raw sub-set of data and the time intervals between each survey point (Table 2), and then examined the power for the same trend with a survey interval of every three years and every year for comparison. *Post-hoc* power analyses typically result in better confidence with a large sample size, and/or small survey intervals with low annual variability and a strong positive or negative trend. This prohibits a stable population from having a high power. Therefore, we used the power of each trend by colony as well as the difference between the 95% confidence interval of that trend to examine the reliability of each trend for each colony.

The *post-hoc* power analysis was applied following procedures in [61–63], using ‘Trends’ (v3.0, Gerrodette and Brandon 2015, https://swfsc.noaa.gov/textblock.aspx?Division=PRD&ParentMenuId=228&id=4740, accessed 1 June 2016). To account for the differences in the number and periodicity of surveys (Table 2), each colony was analysed individually by defining the total duration of the study (i.e. 1986 to 2015), the number of surveys (*n*) and the survey interval. Inputs from the corresponding GLM regression results for that colony were included i.e. the rate of change or slope of the regression line; the coefficient of variation (*CV = 1/√Theta*), which provides a measure of the precision; the significance level (probability of Type 1 error); and the power level or probability of detecting a true change in population (1 – probability of Type 2 error). One of these five parameters could be estimated when the others were provided.

The power analysis variance structure was set to ‘constant’ because in a fur seal population, CV can be expected to increase with abundance (see Hatch 2003 for a detailed explanation). We also set the significance level to 0.05 using a two-tailed test and for the type of change, selected an exponential model (from two choices exponential or linear). We then selected either a positive or negative trend as applicable. The minimum number of samples (or surveys) required was assessed at power = 0.8. The program ‘Trends’ would not accept an input of zero slope (β year = 0.0), therefore where this occurred, 0.01 was used. Power was not calculated for trends with *θ* > 5,000 because these trends were produced from minimal data and the power of such trends could not be calculated.

## Results

### Species-wide census of pup abundance

The 2013 census of the AFS resulted in a total of 14,248 live pups at 20 colonies (Tables 2 & 3). This is an underestimate of species-wide pup abundance because The Skerries (2,254 live pups) and Maatsuyker Island (13 live pups) were surveyed in 2014 and their numbers not included in the 2013 census (Table 2). We combined these results from 2014 with the 2013 census results to enable a complete comparison across censuses (Table 3). The 2013 census detected a reduction from the 2007 census of 21,387 live pups at 20 colonies (Table 3). Several colonies experienced a reduction in pup abundance in the 2013 census for the first time since monitoring began, with colonies of greater pup abundance (>1500 pups: Lady Julia Percy Island, Seal Rocks, Kanowna Island, The Skerries and Judgment Rocks) showing a negative percentage change in pup numbers compared to the 2007 census (Fig 1, Table 3). Reid Rocks, also with >1500 pups showed a large increase compared to 2007 (Fig 1, Table 3). The largest percentage changes in pup number occurred in smaller colonies (+60 at Cape Bridgewater and -18 at Tenth Island, Fig 1, Table 3). Two colonies, Walker Island and Wender Island, both with one pup in 2007, were not visited in 2013 [36]. Three colonies were new additions to the known breeding sites, Walker Island and Baudin Rocks in South Australia and Sloop Rocks in Tasmania (Fig 1). Williams Island extended the known breeding range of the AFS to the west of their former range. The standard errors for the live pup estimates show a high level of precision for the estimates in 2013 (Table 3).

**Table 3:** Estimated Australian fur seal pup numbers from the 2013 census, compared with previous censuses in 2002 and 2007 [39, 54]. Note the 2002 Kanowna Island pup estimate is a direct count and therefore not included in the trend analysis that is based on capture-mark-resight results (CMR). Where no standard error (s.e.) is reported, single direct counts were performed. Data for The Skerries and Maatsuyker Island were obtained in 2014 not during the 2013 census, but are provided here for comparison with previous censuses. 2013 Census results without the inclusion of these two sites results in 14,248 live pups.

Site	2013 census			Previous census		% Change per annum 2007–2013
	No. resight estimates	Pups marked CMR	Live pups (s.e.)	2007 Live pups (s.e.)	2002 Live pups (s.e.)	
VICTORIA						
Seal Rocks	6	1787	4,092 (38)	5,660 (83)	4,882 (51)	-5.3
Lady Julia Percy Is	6	1449	2,659 (16)	5,574 (73)	5,899 (43)	-11.6
Kanowna Is	25 ^B^	1110	2,429 (27)	2,913 (110)	2301 (21) ^A^	-3
The Skerries	4	924	2,254 (33)	2,705 (31)	2,486 (41)	-3
Rag Is			295	277 ^A^	30	1.1
Cape Bridgewater			120	7 ^A^	7	60.6
**SUB-TOTAL**			**11,849**	**17,136**	**15,605**	**-6.0**
TASMANIA						
Judgement Rocks	6	558	1,710 (24)	2,387 (75)	2,427 (100)	-5.4
Reid Rocks			1,570 (60)	395 ^C^	259 (34) ^C^	25.9
Moriarty Rocks			486 (09)	598 (09)	1,007 (08)	-3.4
West Moncoeur			256 (03)	204 (06)	257 (06)	3.9
Wright Rocks			187 (02)	130 (01)	5	6.2
Double Rocks			157 (02)	51	-	20.6
Tenth Is	12	94	138 (04)	448 (20)	124	-17.8
Bull Rock			21	7	7	20.1
Sloop Rocks			16	-	-	-
Iles des Phoques			10 ^A^	0	1	46.8
Maatsuyker Is			13 ^A^	1 ^A^	-	44.3
**SUB-TOTAL**			**4,564**	**4,221**	**4,087**	**1.3**
NEW SOUTH WALES						
Montague Is	7	18	19 (0.3)	2	1	45.5
**SUB-TOTAL**			**19**	**2**	**-**	**45.5**
SOUTH AUSTRALIA						
North Casuarina Is	6	35	75 (3.2)	29 (1.3)	-	17.2
Williams Is			2 ^A^	-	-	-
Baudin Rocks			6 ^A^	-	-	-
Cape Gantheaume			1 ^D^	0	-	-
**SUB-TOTAL**			**84**	**29**	**-**	**17.2**
			***16*,*516***	***21*,*388***	***19*,*692***	***-4*.*2***

^A^ Direct count

^B^ At Kanowna Is, four, eight and 25 resight estimates were performed at two, one and five sub-locations respectively

^C^ Counts differ from Kirkwood et al. (2010) and do not include any multiplicative factors: data confirmed by S.Thalman, DPIPWE, no s.e. available for 2007

^D^ Incidental observation and a possible hybrid with *Arctocephalus forsteri*, one pup also seen in 2012–13, and a hybrid identified in 1995 [55]

### Temporal trends in pup abundance

**Fig 2:**
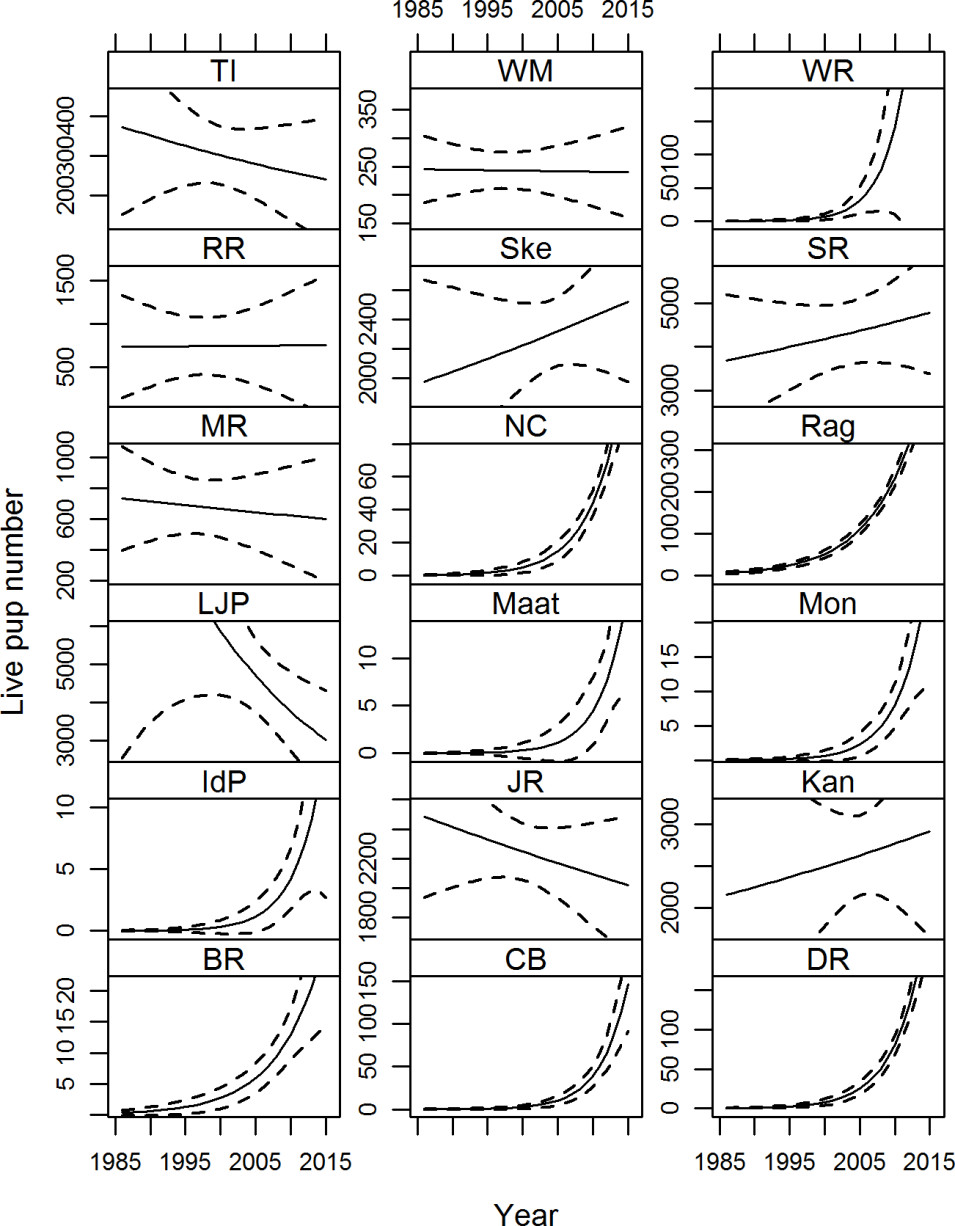
Smoothed predicted curves fitted to raw counts of Australian fur seal pups at breeding colonies in south-eastern Australia, estimated using Generalised Linear Models with negative binomial distributions. Colony abbreviations are: Tenth Island (TI), West Moncoeur (WM), Wright Rocks (WR), Reid Rocks (RR), The Skerries (Ske), Seal Rocks (SR), Moriarty Rocks (MR), North Casuarina (NC), Rag Island (Rag), Lady Julia Percy Island (LJP), Maatsuyker Island (Maat), Montague Island (Mon), Iles des Phoques (IdP), Judgment Rocks (JR), Kanowna Island (Kan), Bull Rock (BR), Cape Bridgewater (CB), and Double Rocks (DR).

According to the trend analysis, ten colonies showed significant changes over the study period (Table 3, Fig 2). However, the degrees of freedom were small and the dispersion parameter (*θ*) was highly inflated for all but three (Lady Julia Percy Island, Cape Bridgewater and Wright Rocks) of these nine colonies (Table 4). The negative binomial can better predict the trend for over-dispersed data (dispersion parameter for Poisson distribution is taken to be “I”), however when the dispersion parameter is highly inflated (e.g. *θ* = > 5000) the result of the GLM is less reliable [58]. At Lady Julia Percy Island, Judgment Rocks and Tenth Island, the large reduction in pup numbers detected in the 2013 census (Table 3) had a strong influence on the trend for those colonies (Fig 2).

**Table 4:** Results of the 2007–08 and 2013–14 Australian fur seal censuses, the associated trends and power analyses. All significant trends were positive with the exception of Lady Julia Percy Island (LJP). Insignificant trends were both positive and negative as shown by *β Year*. The dispersion parameter is identified by theta (*θ)*.

	Negative Binomial GLM									Power analysis using ‘Trends’ program					
*Col*	*df*	*β Year*	*Intercept*	*z*	*P*	*- CI*	*+ CI*	*Dev Exp*	*θ*	*CV*	*MA*	*Trend duration* *(years)*	*Power* *(int = raw data)*	*Power (int = 3)*	*Power (int = 1)*
Sites ordered by -/+ significant trends, then ordered by *df* and smallest CI															
LJP	3	-0.04	97.52	-2.26	0.024	-0.09	0.00	51.07	23.13	0.208	4750	15	0.23	0.91	1.00
WR	8	0.28	-550.33	6.50	0.000	0.20	0.36	87.41	1.96	0.693	37	24	1.00	1.00	1.00
CB	5	0.27	-530.91	7.73	0.000	0.20	0.34	93.51	11.59	0.294	63	21	1.00	1.00	1.00
BR	4	0.16	-313.58	4.91	0.000	0.10	0.22	85.31	>10,000	0.003	8	15	-	-	-
Mon	4	0.25	-505.27	4.53	0.000	0.15	0.37	88.71	>5,000	0.013	5	21	-	-	-
IdP	4	0.26	-519.82	3.32	0.001	0.13	0.45	80.19	>10,000	0.005	2	13	-	-	-
Rag	3	0.15	-297.11	17.89	0.000	0.13	0.17	74.39	>10,000	0.001	151	21	-	-	-
NC	3	0.22	-431.97	7.58	0.000	0.16	0.28	93.53	>10,000	0.004	45	17	-	-	-
DR	2	0.23	-463.48	10.45	0.000	0.19	0.28	95.26	>10,000	0.002	69	17	-	-	-
Maat	2	0.27	-544.45	2.60	0.009	0.17	0.46	99.85	>10,000	0.003	5	25	-	-	-
Sites with insignificant trends ordered by “Power (*int = raw data*)”															
MR	9	-0.01	20.10	-0.39	0.699	-0.04	0.03	1.25	5.74	0.417	681	25	0.25	0.23	0.55
JR	8	-0.01	22.01	-0.97	0.334	-0.02	0.01	8.48	60.14	0.129	2247	20	0.22	0.25	0.60
WM	9	0.00	6.94	-0.08	0.935	-0.02	0.02	0.06	24.78	0.201	243	25	0.18	0.17	0.41
TI	7	-0.02	35.95	-0.76	0.447	-0.06	0.03	5.51	8.45	0.344	303	20	0.15	0.17	0.40
SR	6	0.01	-6.23	0.74	0.457	-0.01	0.03	6.27	25.95	0.196	4372	23	0.13	0.16	0.31
Ske	4	0.01	-9.00	0.90	0.366	-0.01	0.03	13.22	78.95	0.113	2310	16	0.12	0.09	0.28
RR	7	0.01	-19.49	0.36	0.719	-0.04	0.07	2.20	1.71	0.765	676	28	0.07	0.11	0.22
Kan	4	0.01	-13.08	0.50	0.620	-0.03	0.06	3.95	23.32	0.207	2642	14	0.06	0.07	0.09

Note: Negative Binomial Generalised Linear Models (GLM) were applied to the raw pup abundance data presented in Table 2; regression results are provided including the percentage deviance explained (*Dev Exp*) and the dispersion parameter (*θ)* of the GLM. Colonies (*Col*) with high Dev Exp and low *θ* (reliable results) are shaded, as are sites with no significant change detected but high power. Power analyses were not performed for sites with unreliable trends (*θ > 5000)*. Power analyses were based on the mean abundance (*MA*) for each colony, the beta value for year (*β Year*, the slope of the trend) and the trend duration (years). *Int = raw data* represents the power analyses performed using the raw data provided in Table 3, including the associated time intervals between surveys (in years); *int = 3* and *int = 1* are the simulated power calculations based on three and one year sampling intervals. Insignificant trends for Moriarty Rocks (MR) and Judgment Rocks (JR) showed high power (>0.20).

### Power to detect changes in trends

The *a-priori* power analysis based on the GLMs identified that when surveying every five years, we would require 15 surveys (75 years) to detect a 30% change in the population with 90% confidence (p<0.10) and 0.80 power. If colonies were surveyed every three years, nine surveys would be sufficient (27 years total).

In this study, the trends with higher power and therefore greater reliability within the parameters defined in the methods were at colonies Lady Julia Percy Island, Cape Bridgewater, Judgment Rocks, Moriarty Rocks and Wright Rocks (Table 4). The high power was caused by different attributes of each trend: for Lady Julia Percy Island, it was the large influence of the 2013 data point and the associated steep decline in the slope; for Cape Bridgewater, it was because the relatively young colony had been in a phase of exponential growth; in the case of Moriarty Rocks, there was a large sample size in terms of number of surveys; and for Wright Rocks, it was because the colony, despite not being a new colony, was in a strong growth phase (Table 4, Fig 2). Furthermore, the relatively wide confidence interval for Wright Rocks (Table 4) is an example of when the confidence interval of a trend may better express the reliability (or lack thereof) of the trend rather than the power. This confidence interval is wide because the two most recent censuses (2007 and 2013) show a strong positive deviation from the previous assessments for the site (1989–2002 in Table 2) that had also been performed more frequently (Fig 2). At Judgment Rocks, the four sequential and similar pup estimates from 1996 to 1999 improved the power of the trend (power = 0.22; -CI, +CI = -0.04, +0.03, Fig 2).

For several colonies that had a greater number of surveys (Table 4, i.e. Seal Rocks, West Moncoeur, Tenth Island), the power of the trends was lower than expected (<0.20). However, the upper and lower confidence interval for colonies Seal Rocks and West Moncoeur were small (Table 4), indicating good reliability in the trends for these colonies (Fig 2). For colony West Moncoeur, the slope of the line was zero and the small upper and lower confidence intervals identified that this trend was reliable (Table 4). Seal Rocks also had a higher confidence (small confidence intervals) in the trend than the colony at Tenth Island and some of the other colonies for which the trends had high power (Lady Julia Percy, Cape Bridgewater, Moriarty Rocks and Wright Rocks); however, Tenth Island had a larger spread of pup estimates over a shorter time period (Fig 2 and Table 2). Power analyses were not performed for any trends with high dispersion parameters (θ > 5,000) because of the small sample size and lack of reliability.

Generally, the power of a trend increased as the interval between estimates decreased (Table 4). Standardising the interval to three years between estimates therefore resulted in increased power, except for those colonies that had intervals between estimates of less than three years, or data clustered over short time periods.

## Discussion

In this paper, we report the first reduction of annual pup production (-4.2% per annum, Table 3) by the AFS since species-wide protection was implemented in 1975. Between 1986 and 2002, growth was sustained at estimated rates >5% per annum resulting in a more than doubling of the pup production [39]. Between 2002 and 2007 pup production appeared to stabilize [36]. Attempting to communicate the reduction in pup numbers for the 2013 census prompted an examination of the capacity of a 5-yearly census to detect meaningful change in the population.

The observed reduction in the total number of live pups in 2013–14 relative to previous years primarily reflected reduced numbers of pups at the largest colonies. Despite the overall reduction in live pup numbers, some colonies showed an increase in numbers and three new colonies were identified (Fig 1). The reduction in pup numbers may indicate that the population has approached a regional carrying capacity, or density-dependant capacity at the established colonies as was speculated by Kirkwood, Pemberton [36], or it could be a first data-point from a sustained and as yet undetected decline. Alternatively, it could simply be because this was an unusually poor period for food availability. Such contrasting interpretations highlight a shortcoming in the ability of a 5-yearly census to quickly identify even gross changes. However, even with more frequent surveys, to gain a detailed understanding of population changes, associated information including demographic and foraging ecology data would be required [4, 14, 33].

### Interpreting changes in pup numbers

Bottom up-effects of environmental variability mediated through prey availability may have caused the reduction in AFS pup numbers in 2013–14. Indeed, food availability for predators in Bass Strait was considered to be low during this time [64, 65]. Seabirds foraging on the shelf of Bass Strait also had poor breeding seasons that year. Large numbers of short-tailed shearwaters (*Ardenna tenuirostris*), which overlap in breeding range with the fur seals, were found dead along the Australian coastline and the ‘wreck’, as such an occurrence is termed, was related to storms and starvation over the expanse of their migration [64]. Breeding success in 2013 was reduced for: short-tailed shearwaters, Australasian gannets (*Morus serrator*) and little penguins (*Eudyptula minor*) in south-eastern Australia [66–68]; little penguins (the number of chicks per breeding female for 2013 was 0.60, compared to the average of 1.08, SD 0.2 from 1997–2012) and crested terns (*Thalasseus bergii*) on Phillip Island (Unpub. data, Phillip Island Nature Parks, Australia); and shy albatross (*Thalassarche cauta*) on Albatross Island in western Bass Strait, where breeding success was only 26%, the lowest since monitoring began in 1989 [65]. At larger fur seal colonies effects of variability in prey resources may be exacerbated because of the increased likelihood of intraspecific competition for resources [28, 69–71]; while we do not have the supporting evidence, this could explain why the drop in live pup numbers were mainly associated with larger colonies such as Lady Julia Percy Island, Seal Rocks, Kanowna Island, and The Skerries.

Kirkman, Yemane [72] also reported varied trends between colonies of the conspecific Cape fur seal (*Arctocephalus pusillus pusillus*) in southern Africa. This included decreases at several of the largest colonies, stability or growth at other colonies and development of new colonies, similar to what has been shown for the AFS in this study, although the latter’s population is only 5% that of the Cape fur seal. Effects (or side-effects) of management, density dependence and shifts in prey distributions have been identified as potential causes of the declines in the case of the Cape fur seal [72].

Prey availability is the most likely regulator of population size for the AFS and while the reduction in pup abundance may be an isolated event, it is the first reduction recorded by censuses that have previously captured population growth. Tenth Island had the highest % change (-17.8% per annum) for all colonies (Fig 1, Table 3), however, this colony is known for highly variable pup estimates because it is low lying and pup numbers are affected by wave wash [29].The reduction in pup numbers at Lady Julia Percy Island in 2013 compared to 2007 (-11.6% per annum) is the next largest reduction in pups. An alopecia syndrome that affects thermoregulation and may reduce female survival in AFSs has been recognised at Lady Julia Percy Island [73] and may have exacerbated the pup reduction at this site. It is thought that this syndrome could be the expression of endocrine disrupting dioxin persistent organic pollutants [74]. Fluctuations in live pup counts can be caused by abnormally high early pup mortality (e.g. due to summer storms) before a census, or a high rate of aborted pregnancies prior to the breeding season, and therefore may not be a good reflection of the breeding population when considered in isolation [29, 75]. In Antarctic fur seals (*Arctocephalus gazella*), pup mortality correlates to colony density because as populations increase and space is less available, more pups may die from being crushed or separated from mothers [76]. However, it is important to appreciate that colony densities for Australian fur seals are far lower than those observed for example, in Cape fur seals and Antarctic fur seals at South Georgia, where density dependent effects on pup mortality are higher [72, 76]. Demographic assessments including counts of dead pups would be needed to differentiate recruitment issues from a short-term reduction in pup production.

### Reviewing the AFS monitoring program

In 2013, we detected the first drop in live pup numbers since the beginning of the monitoring program in the 1960s. Unfortunately, lack of corroborative data prevents us from identifying the cause of this drop. To investigate the reasons behind population change, information relating to diet and demography including seal health, density and age structure are required. [32, 69, 76, 77]. However, even with such information, it can be difficult to tease apart the drivers of population change. For example, competition can act on a population of high density to reduce population growth, and also Allee effects can work the other way when there are benefits to living in group such as predator detection and avoidance [71, 78].

Oceanographic influences and food supply will vary across the range of the AFS [79–82], likely affecting diet and demography [83]. The diet of the Australian fur seal is being monitored [30], there has also been some research into disease, pup body condition and health [73, 74, 84]. Future research needs to combine these projects temporally and spatially so that we can interpret the changes we are observing in the ecosystem. Increasing the parameters to be measured without reducing the sites being visited will increase the cost, effort and logistics for the monitoring program. It is therefore necessary to prioritise several sites for more intensive monitoring.

For Australian fur seals, ecological differences exist between colonies in different locations, such as the influence of different current and upwelling systems, proximity to urbanization and varying land-use practices as well as variation in diet, and demography (Fig 1, Table 5). Also, recently established colonies and those on the boundaries of the range may exhibit different demographic parameters and different trends than longer established colonies in the centre of the range [85]. These are all factors that may influence the contrasting trends that were evident between colonies (Tables 4 and 5, Fig 2). Several management-agencies obtain and use the data, each with their own challenges and objectives. This adds additional complexity and makes it difficult to prioritize colonies to improve efficiency. However, in an attempt to do so, we have roughly grouped the colonies according to their attributes and trends (Table 5). It may be possible to select one colony from each group to represent those attributes as done by Kirkman, Oosthuizen [2]. While outside the scope of this paper and because of current data deficiencies, a decision-theoretic framework could be applied to assist with designing an improved monitoring program for the Australian fur seal [86].

**Table 5:** Results of the 2013 and 2007 Australian fur seal censuses, ordered by group and then number of live pups in 2013. Colonies are grouped by similar attributes of capacity, major regional feature and trend. Potential impacts and threatening processes are identified where they exist. These attributes may be taken into account for planning and prioritising monitoring in the face of logistic or funding constraints. Information was obtained from the literature [29, 73, 79, 80, 103–107] and from results of this study. Group refers to colonies with similar abundance and trends in 2013. Storm mortality refers to storm-induced pup mortality that can cause large fluctuations in estimates. Trend summarises results from Table 4 and Fig 2. The state provides the region of management–Victoria (VIC), Tasmania (TAS), New South Wales (NSW) and South Australia (SA).

Group	Colony	Near capacity	Regional feature	Potential impacts	Trend (Fig 2)	State
1	Seal Rocks	perhaps	Close to Port Phillip Bay and the city of Melbourne and Western Port	Oil spill	Slowing growth	VIC
1	Kanowna Is.	uncertain	Wilson’s Promontory and East Australian Current (EAC)	Overlap with Danish seine fishery	Growth	VIC
1	The Skerries	uncertain	EAC	Storm mortality; Overlap with Danish seine fishery	Growth	VIC
2	Lady Julia Percy	perhaps	Bonney Upwelling	Unique disease; Overlap with trawl fisheries	Decline	VIC
3	Judgement Rocks	uncertain	Eastern Bass Strait, EAC		Decline	TAS
3	Moriarty Rocks	uncertain	Eastern Bass Strait, EAC	Storm mortality	Decline	TAS
3	Tenth Is.	Yes	Eastern Bass Strait, EAC	Storm mortality	Decline	TAS
4	Reid Rocks	uncertain	West Tasmanian Upwelling	Storm mortality; Overlap with trawl fisheries	Growth	TAS
4	Rag Is.	no	EAC		Logistic growth	VIC
4	Wright Rocks	no	EAC		Logistic growth	TAS
4	Double Is.	no	NE Tasmanian upwelling		Logistic growth	TAS
4	Cape Bridgewater	no	Bonney Upwelling		Logistic growth	VIC
5	West Moncoeur	uncertain	Eastern Bass Strait, EAC		Stable	TAS
6	North Casuarina	no	Localised Upwelling		Logistic growth	SA
6	Bull Rock	no			Logistic growth	TAS
6	Montague Is.	no	Edge of range		Logistic growth	NSW
7	Sloop Rocks	no		Overlap with trawl fisheries; Aquaculture interaction	Identified 2014–15	TAS
7	Maatsuyker	no	Edge of range	Overlap with trawl fisheries; Aquaculture interaction	No trend	TAS
7	Illes des Phoques	no		Aquaculture interaction	No trend	TAS
7	Baudin Rocks	no	Bonney Upwelling		Identified 2014–15	SA
7	Williams Is.	no	Edge of range		Identified 2014–15	SA
7	Cape Gantheaume	perhaps with *A*. *forsteri*				SA

The power analysis of the trends in this study shows that we could obtain more reliable trends by sampling more frequently than every five years. For species with extensive ranges that are spatially complex, increased effort may be necessary to obtain reliable trends; this is even more pronounced when populations reach carrying capacity and abundance estimates fluctuate around a certain level. However, increased sampling effort can lead to increased disturbance and may not be logistically possible. The Trilateral Working Group (TWG) comprising representatives from The Netherlands, Germany and Denmark performed a power analysis of their monitoring program for the harbour seal (*Phoca vitulina*) in the Wadden Sea [17]. After 45 years of annual monitoring, this group aimed to reduce sampling effort to every second or third year. Contrary to what they had hoped, their annual program lacked sufficient power to rely on the results of their trends; therefore they maintained the annual monitoring program. For the Australian fur seal, the best strategy is to balance the spatial and temporal scale of the monitoring program with increasing the parameters being sampled. We may have to accept a level of uncertainty in the trends analysis until we have more data while we incorporate those parameters that will help us to understand any changes observed in the population.

The ideal paradigm for the long-term monitoring of populations is adaptive monitoring [21], a paradigm that aligns with adaptive management and structured decision-making [87, 88]. Adaptive management aims to reduce uncertainty, incorporate resilience and accrue information to inform future decision making thereby improving the outcomes of management actions [88]. Adaptive or dynamic monitoring relies on a robust conceptual model of the ecosystem under study and aims to learn from that environment and account for uncertainty in the measurements and temporal variation [89]. It incorporates clear objectives, tractable questions, good statistical design, and an ability to alter the program in response to ecosystem changes, technological advances, and altered information requirements [21, 90]. Importantly, collaboration between scientists, resource managers and policy makers also ensures that the program remains relevant [21, 90, 91]. Together, these components readily differentiate the paradigm from *ad hoc*, reactive, or surveillance monitoring [88, 92, 93].

An improved monitoring program would be to survey selected sites for live and dead pups, pup body condition and health, and density of seals at least every three years and perform a range-wide census every 10 years. As a very basic requirement, dead pups should be counted at the same time as live pups to help elucidate changes in pup survival that could be related to density, the ability of mothers to provision pups, or disease [2, 70, 94, 95]. Importantly, the method of survey needs to be consistent for each site over time. Regular assessments of the program should be performed to ensure goals are being achieved. After the census, the program should be reviewed and sites prioritised for the next 10 years (for an example of a similar sampling regime, see Wege et al 2016 [14]). This approach follows the adaptive monitoring paradigm because it allows for review and change. It incorporates new information and allows changes to the sites of focus, the frequency of monitoring, the parameters being measured and the time frames of surveys. However, this monitoring program does not identify new, establishing, colonies, which is important for monitoring changes in distribution. Periodical aerial surveys may be the most cost-effective method for detecting the colonization of new sites.

There is great potential for using remote piloted aircraft (RPAs) to perform surveys as the technology becomes more accessible and cost effective [96–100]. The major benefits of using RPAs is the reduced effort and cost compared to a CMR (for example), reduced disturbance to the seals, provided height limits are tested, and the potential for an increase in the frequency of surveys as a result. Clearly, if the method at a colony is going to change, it is vital that the estimates be calibrated [101]. RPAs also allow the long-term retention of images that can be revisited for further research and the ability to monitor abundance and density of all age classes (adult females, adult males, sub-adult males, juveniles ~1–3 years of age and pups) [102]. Such information can be used to better measure changes in abundance.

### Management considerations

It is important that a revised monitoring program provides satisfactory outputs for natural resource managers and policy makers. The AFS was harvested to near extinction in the early 1800s, but is now a nationally protected species under the *Environment Protection and Biodiversity Conservation Act* (EPBC Act 1999) and considerable importance has been attached to monitoring its population recovery and responses to management approaches. Abundance data of the population are also used in ecosystem models to understand complex relationships between wildlife and commercial species and advise fisheries managers to aid effective decision making [1, 108]. Abundance data associated with known locations such as seal colonies are useful for responding to emergency situations such as identifying the impacts of oil spills on affected wildlife [26, 109]. Furthermore, spatial abundance data and knowledge of population trends enables managers to maintain updated protected areas, and facilitate and/or mitigate economically important projects, such as natural resource extraction, shipping routes and tourism ventures that seek to utilise a wildlife resource [109, 110].

Finally, population information can have localised relevance. For example, in the state of Tasmania (Fig 1) AFSs interact with the salmonid aquaculture industry, predating fish and damaging property: costing the industry an estimated $AUS 1000 per tonne of salmon produced [111, 112]. While the rate of seal interactions varies seasonally there has been a noted increase in the number of seals interacting each year since 1990 [112]. An understanding of fur seal population trends in association with an assessment of individual seal behavior at the aquaculture site (residency rate at farm site, return rate following translocation, estimates of known seals to be interacting) are critical to understanding whether the rate of interaction is driven by intrinsic population factors or individual interaction frequency [25]. The increasing development of this industry throughout southeast Tasmania also affects the haul-out distribution of the AFS and may influence the establishment of new colonies at locations such as Iles des Phoques.

### Conclusion

The monitoring of the AFS between the 1970s and 2013, incorporating periods of annual estimates at some locations, opportunistic surveys at others and three population-wide surveys, has effectively recorded change during a growth period for the seals. Into the future, however, continuation of current strategies may not reliably detect density dependent regulation of the population, or allow rapid recognition between, for example, an anomalous result and a decline.

It is understood that there are multiple reasons for monitoring a colony, which will largely be driven by the question of interest. This paper highlights key parameters that need to be measured including at the least, pup mortality and density, while also providing information to assist with the prioritising of colonies. Here we have provided an example of why monitoring programs should be assessed regularly, with the aim of improving them at regular intervals. This maximises the chance that the monitoring program is achieving its goals and responding to change. Independent research programs investigating the diet, health and pup trends need to be coordinated, with the addition of demographic information to understand this drop in live Australian fur seal pups.
